# The science of sex and gender in human health: online courses to create a foundation for sex and gender accountability in biomedical research and treatment

**DOI:** 10.1186/s13293-016-0100-z

**Published:** 2016-10-14

**Authors:** Jennifer L. Plank-Bazinet, Annie Sampson, Leah R. Miller, Emmanuel O. Fadiran, Deborah Kallgren, Rajeev K. Agarwal, Whitney Barfield, Claudette E. Brooks, Lisa Begg, Amy C. Mistretta, Pamela E. Scott, Janine Austin Clayton, Terri L. Cornelison

**Affiliations:** 1 National Institutes of Health Office of Research on Women’s Health, 6707 Democracy Blvd, Suite 400, Bethesda, MD 20817 USA; 2 Food and Drug Administration Office of Women’s Health, 10903 New Hampshire Avenue, W032-2333, Silver Spring, MD 20993 USA

**Keywords:** Sex, Gender, Continuing medical education, Continuing nursing education, Continuing pharmacy education, Disease-specific sex and gender differences, Disease manifestation and outcomes

## Abstract

**Background:**

Sex and gender differences play a significant role in the course and outcome of conditions that affect specific organ systems in the human body. Research on differences in the effects of medical intervention has helped scientists develop a number of sex- and gender-specific guidelines on the treatment and management of these conditions. An online series of courses, “The Science of Sex and Gender in Human Health,” developed by the National Institutes of Health Office of Research on Women’s Health and the U.S. Food and Drug Administration Office of Women’s Health, examines sex and gender differences and their implications. Thus far, three online courses have been generated. The first course offers an overview of the scientific and biological basis for sex- and gender-related differences. The second course is focused on disease-specific sex and gender differences in health and behavior and their implications. Finally, the third course covers the influence of sex and gender on disease manifestation, treatment, and outcome.

**Methods:**

Data were obtained using website analytics and post-course surveys.

**Results:**

To date, over 1000 individuals have completed at least one course. Additionally, 600 users have received continuing education credit for completing a course in the series. Finally, the majority of respondents to the online course survey have indicated that the courses considerably enhanced their professional effectiveness.

**Conclusions:**

“The Science of Sex and Gender in Human Health” online courses are freely available sources of information that provide healthcare providers and researchers with the resources to successfully account for sex and gender in their medical practice and research programs.

## Background

Sex and gender differences play a significant role in the course and outcome of conditions that affect specific organ systems in the human body [1, 2]. Research on differences in the effects of medical interventions has helped scientists develop a number of sex- and gender-specific guidelines on the treatment and management of these conditions.

In an attempt to disseminate the wealth of information related to sex- and gender-based health differences, The National Institutes of Health (NIH) Office of Research on Women’s Health (ORWH) and the Food and Drug Administration (FDA) Office of Women’s Health (OWH) developed a series of online courses titled “The Science of Sex and Gender in Human Health.” These courses examine sex and gender differences and their implications [3]. There are a total of three courses available. The first was launched in 2006, followed by the second and third phases in 2010 and 2014, respectively.

The series builds upon the NIH- and FDA-funded 2001 Institute of Medicine report, “Exploring the Biological Contributions to Human Health: Does Sex Matter?” [2]. This report was critical in highlighting the need to consider sex (being male or female, according to reproductive organs and chromosomes) and gender (one’s sense of self as male or female in society) as variables in human health and unequivocally indicated that sex *does* matter when developing individualized treatments. However, despite the findings of the report, many institutions of higher education have struggled to fully incorporate sex and gender into their curricula [4]. In September 2012, the Mayo Clinic hosted a workshop to develop strategies to include the concepts of sex and gender in health professional curricula [4]. One proposed strategy was the development of digital training tools.

Since the 2001 Institute of Medicine report, there has been an ever growing body of research that highlights the importance of considering influences of sex and gender. For example, low-dose aspirin has different preventive effects in men and women. In women, aspirin reduces risk of ischemic stroke, whereas in men, low-dose aspirin therapy reduces risk of heart attack [5, 6]. Cholesterol plaque in women may not build up into major artery blockages but instead spreads evenly throughout the artery wall. This means that artery blockages can be more difficult to diagnose in women, who may not have outright symptoms, but are still at a high risk for a heart attack [7]. The clinical implication of this knowledge is underscored by the fact that women who believe they are at risk for developing cardiovascular disease (CVD) are likely to seek treatment or take action to improve their health; however, many women are unaware of the prevalence of CVD in women [8]. Women also experience higher rates of adverse drug reactions than men [9]. Zolpidem, used to treat insomnia, has a lower recommended initial dose for women due to the risk of next-morning impairment resulting from delayed drug clearance in women [10, 11]. Sex differences in drug toxicity and safety further highlight the need to consider sex and gender influences. From 1997 to 2000, 8 out of 10 drugs that were withdrawn from the market by the FDA had greater health risks in women than in men [12]. These are but a few of many examples that demonstrate the importance of considering sex and gender in research and practice. The online courses described here provide an ideal tool to build a foundation of knowledge of the concepts of sex and gender and how they influence human health and the manifestation and treatment of disease.

The courses were designed while considering the theory of adult learning [13, 14]. Andragogy, the instruction of adults, differs from pedagogy, the instruction of children in several distinct ways. Six basic assumptions were made when developing a model of adult learning: (1) adults need to know why they are learning something; (2) as people age they develop a more self-directed personality; (3) experiences gained throughout life are valuable; (4) adult learners are interested in things relevant to their lives; (5) adults are more problem-centered than young learners; and (6) adults are intrinsically more self-motivated [13]. Each of these assumptions was factored into course design. Users who are completing the courses are self-directed and self-motivated and opted to complete the course based on a need to enhance their professional capabilities or because there is a desire to learn about the topic. The topic is generally relevant to the careers of the users, and the experience the user has prior to completing the courses results in a more enriching experience.

The course steering committee also considered best practices in designing self-paced, online courses. One key focus was usability, to assure the learners could effectively take advantage of the software and platform [15]. Another key component of maximizing the user experience is consulting experts and garnering expertise while designing and writing each lesson [15]. The steering committee collaborated with outside experts from multiple medical and scientific fields from across the country when designing each of the lessons in the courses to assure that users were learning state-of-the-science content.

Although “The Science of Sex and Gender in Human Health” courses are designed primarily for researchers, healthcare providers, educators, and students in health professional schools, the courses are free and available to the public. Each course in the series is self-paced and consists of five to six lessons. Continuing education credit is available for eligible users, while other users who complete the course can receive a certificate of completion. An online quiz follows each lesson, and a minimum of 70 % accuracy is required for successful completion. At the end of each course is an online survey to provide feedback on the course content and effectiveness.

To date, the courses have been completed 1223 times and 600 registrants have received continuing education credit. Post-course surveys indicate that the majority of respondents felt the courses met their objectives. Here, we provide an overview of the courses, present data on course completion and user satisfaction, and preview future modifications to the courses.

## Methods

The information about course users and user satisfaction was derived from course registration information and post-course surveys. The additional data collected were from trend analyses and included the number of individuals who viewed the course, number of individuals who completed the course, and length of time spent on the site. Digital analytics were used to pull and evaluate data.

### Course descriptions

**Fig. 1:**
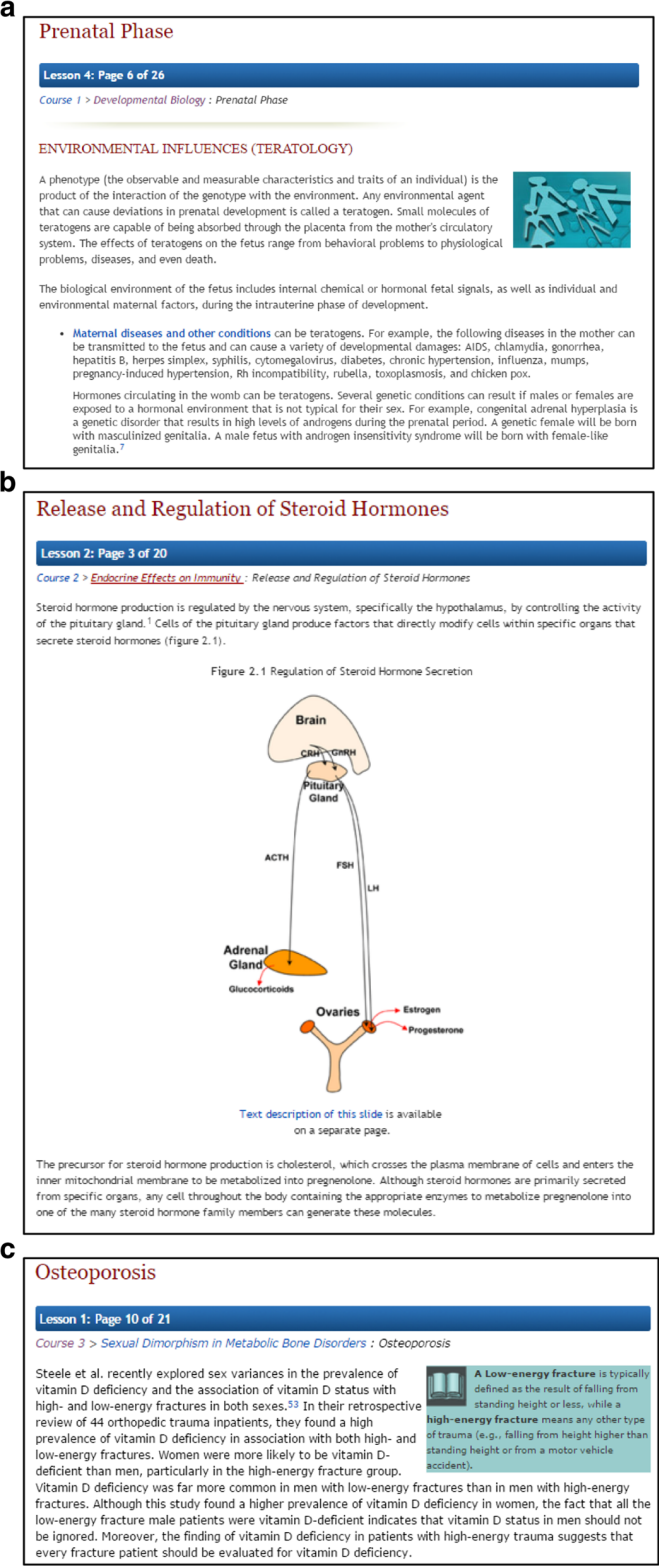
Screenshots of course lessons. Screenshots from course 1 (**a**), course 2 (**b**), and course 3 (**c**) were captured to illustrate the format of the lessons. A different presentation style was highlighted in each panel

The courses are primarily text-based, with data presented in tables and figures. Additionally, key terms and concepts are defined in colored text boxes in the margins. Screenshots from each course are depicted in Fig. 1. Although different elements are highlighted in the figure, all of the courses used a similar format for instruction. The screenshot from course 1 shows a page that is largely text-based, while the page excerpted from course 2 contains a figure, and the page from course 3 contains a text box defining key terms (Fig. 1). In the current form, the courses work most effectively on a desktop or laptop computer, rather than a mobile device.

While the instructional format of the three courses is the same, the format of the quizzes differs. The quizzes in course 1 consist of completion questions where the user selects an answer from a bank of short phrases. In course 2, the quizzes also consist of completion questions, but the answers contain only one or two words. Finally, the format of the quizzes in course 3 are multiple choice. The format of the quizzes evolved over time to be more challenging for the users, yet enhances the ease of use. The shorter answers and multiple choice format, while containing more challenging questions, are more amendable to an online format than answers in the form of short phrases. Given that knowledge is cumulative, quiz difficulty increased as the courses progressed, but the format was made to be less cumbersome for balance. For all of the courses, users must complete at least 70 % of the questions correctly, and the quizzes can be taken an unlimited number of times.

The first course in the series provides a general overview of the topic and lays a foundation for the second and third courses. Each course consists of 5 or 6 lessons (Table 1), and each lesson takes approximately 1 h to complete. During each lesson, the user reads the content and does not interact with the software until the quiz. While the courses and lessons were designed to be taken sequentially, completing individual lessons may also be beneficial to the participant. In this section, we have highlighted the key points, lessons, and objectives of each of the three courses.

**Table 1:** Course and lesson titles

Course 1: Basic Science and the Biological Basis for Sex- and Gender-Related Differences	Course 2: Sex and Gender Differences in Health and Behavior	Course 3: The Influence of Sex and Gender on Disease Expression and Treatment
Launch date: June 2006	Launch date: October 2010	Launch date: July 2014
Lesson 1: understanding the importance of sex and gender in biomedical research	Lesson 1: clinical research methodology	Lesson 1: sexual dimorphism in metabolic bone disorders
Lesson 2: legislative process framework	Lesson 2: endocrine effects on immunity	Lesson 2: cardiovascular disease in women: a focus on heart failure
Lesson 3: cell physiology	Lesson 3: drug therapeutics during pregnancy	Lesson 3: sex and gender differences in pulmonary function and health
Lesson 4: developmental biology	Lesson 4: understanding the importance of sex and gender in mental health	Lesson 4: the neural basis of sex differences in pain
Lesson 5: pharmacodynamics and pharmacokinetics	Lesson 5: autoimmunity, autoimmune disease, and sex bias	Lesson 5: sex differences in substance abuse and treatment
Lesson 6: clinical applications of genomics	Lesson 6: sex and gender differences in irritable bowel syndrome	

#### Course 1: Basic Science and the Biological Basis for Sex- and Gender-Related Differences

The first course in the series lays a general foundation for understanding sex- and gender-based differences in clinical practice and research, “Basic Science and the Biological Basis for Sex- and Gender-Related Differences.” It offers users a basic overview of the major physiological differences between the sexes, their influence on illness and health outcomes, and their implications for policy, biomedical research, and health care. The lessons comprising this course range from policy implications of inclusion of women in clinical trials to basic biological influences and differences between males and females (Table 1). The objectives of the first course include understanding key policies related to inclusion of women in clinical trials, assessing the design of research studies to detect sex and gender differences, describing the scientific basis of known sex and gender differences, and identifying known sex and gender differences with regard to disease and treatments.

#### Course 2: Sex and Gender Differences in Health and Behavior

The second course, “Sex and Gender Differences in Health and Behavior,” examines disease-specific sex and gender differences in health and behavior and their implications. This course applies the basic concepts demonstrated in the first course to specific conditions and organ systems where sex and gender influences play a significant role. It also presents a research methodology framework to help researchers, clinicians, and students incorporate sex and gender differences into study designs. The lessons in this course include a wide variety of topics including therapeutics during pregnancy, the influence of sex and gender in mental health, and sex biases in autoimmunity and autoimmune disease (Table 1). The objectives achieved in the second course include recognizing study designs to address sex and gender differences, understanding how the nervous and endocrine systems regulate steroid hormones, identifying symptoms of mood disorders, understanding challenges in drug therapeutics during pregnancy, and understanding the difference between autoimmunity and autoimmune disease.

#### Course 3: The Influence of Sex and Gender on Disease Expression and Treatment

The third course, “The Influence of Sex and Gender on Disease Expression and Treatment,” examines the influence of the differences between women and men on disease manifestation, treatment, and outcomes. This course provides participants with an overall understanding of how sex and gender affect the function of select organ systems, disease progression, and treatment options. Lessons in course 3 include cardiovascular disease in women, sex and gender differences in pulmonary function and health, and sex differences in substance abuse and treatment, among others (Table 1). Participants of the third course should be able to identify sex and gender differences in the pathophysiology of the diseases covered in the course, describe how sex differences may affect treatment options, recognize challenges of performing research on major organ systems in both sexes, and explain sex differences in response to substance abuse and treatment.

Participants who complete all three courses will gain ample knowledge for considering the influence of sex and gender differences in the clinic and designing experiments that strategically account for sex and gender as key variables.

## ﻿Results

### Website metrics

**Fig. 2:**
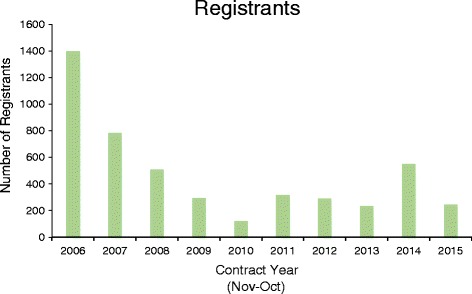
Number of course registrants. The number of course registrants per year is depicted in the graph. The courses were launched in June 2006, October 2010, and July 2014

Since the launch of the courses in 2006, 4744 users have registered for the website (Fig. 2), with 2006 having the greatest number of registrations (1396). Subsequent years have had relatively steady levels of registration, with slight increases in 2011 and 2014, corresponding to the launch of courses 2 and 3, respectively (Fig. 2).

During 2015 (data not shown), there were 2.6 million page views and 75,509 visits by 27,062 different visitors. Furthermore, 69 % of the visitors in 2015 were new to the site. On average, visitors to the website in 2015 spent 35 min and 38 s on the website. Finally, the majority of visitors to the website used a desktop or laptop; however, throughout 2015, there was a steady increase in the number of visitors using mobile devices such as smartphones or tablets.

### Course participants

In order to receive credit for completion of each course, a participant must complete all of the lessons, receive a score greater than 70 % on all of the lesson exams, and complete the course survey. As of October 2015, 991 participants completed all of the course 1 lessons and survey, 199 participants completed all of the course 2 lessons and survey, and 33 participants completed all of the course 3 lessons and survey (Table 2).

**Table 2:** Individual lesson completion from course inception

	Lesson 1	Lesson 2	Lesson 3	Lesson 4	Lesson 5	Lesson 6	Course survey
Course 1: Basic Science and the Biological Basis for Sex- and Gender-Related Differences	2129	1518	1335	1240	1150	1120	991
Course 2: Sex and Gender Differences in Health and Behavior	274	264	250	254	249	247	199
Course 3: The Influence of Sex and Gender on Disease Expression and Treatment	49	45	43	42	43	N/A	33

Many of the participants who complete the course seek continuing education credit. As of October 2015, 600 participants had completed a course to gain continuing education credit (Table 3). Of these participants, the majority (555) obtained continuing medical education (CME) credit for physicians, 39 obtained continuing nursing education (CNE) credit, and 6 obtained continuing pharmacy education (CPE) credit. Another 623 participants successfully completed a course but were not seeking continuing education credit.

**Table 3:** The number of participants who completed a course since its inception or in 2015

	Course 1: Basic Science and the Biological Basis for Sex- and Gender-Related Differences	Course 2: Sex and Gender Differences in Health and Behavior	Course 3: The Influence of Sex and Gender on Disease Expression and Treatment	Total
CME for physicians (2015)	476 (10)	65 (5)	14 (9)	555 (24)
CNE for nurses (2015)	15 (2)	15 (4)	9 (6)	39 (12)
CPE for pharmacists (2015)	4 (1)	1 (1)	1 (1)	6 (3)
Not for credit (2015)	496 (7)	118 (5)	9 (5)	623 (17)
Total (2015)	991 (20)	199 (15)	33 (21)	1223 (56)

During 2015, 24 users completed a course for CME credit, 12 users completed a course for CNE credit, and 3 users completed a course for CPE credit (Table 3). Another 17 users completed a course, but they were not eligible for or interested in continuing education credit. Moreover, in 2015, a total of 20 users completed course 1, 15 users completed course 2, and 21 users completed course 3 (Table 3). In 2014 (data not shown), 59 users completed a course for CME credit, 20 users for CNE credit, and 3 users for CPE credit, while 110 users completed a course but were not eligible for or interested in continuing education credit.

### Participant satisfaction

Upon completion of the courses, users are required to complete an evaluation form in order to receive continuing education credit or a completion certificate. The purpose of the evaluation is to assess a user’s level of satisfaction with each course and to determine if the course met its objectives. The overwhelming majority of the participants indicated that the courses considerably or completely enhanced their professional effectiveness. For course 1, 76 % of the respondents gave this favorable response, while 83 and 77 % of the respondents said the same for courses 2 and 3, respectively (data not shown).

**Fig. 3:**
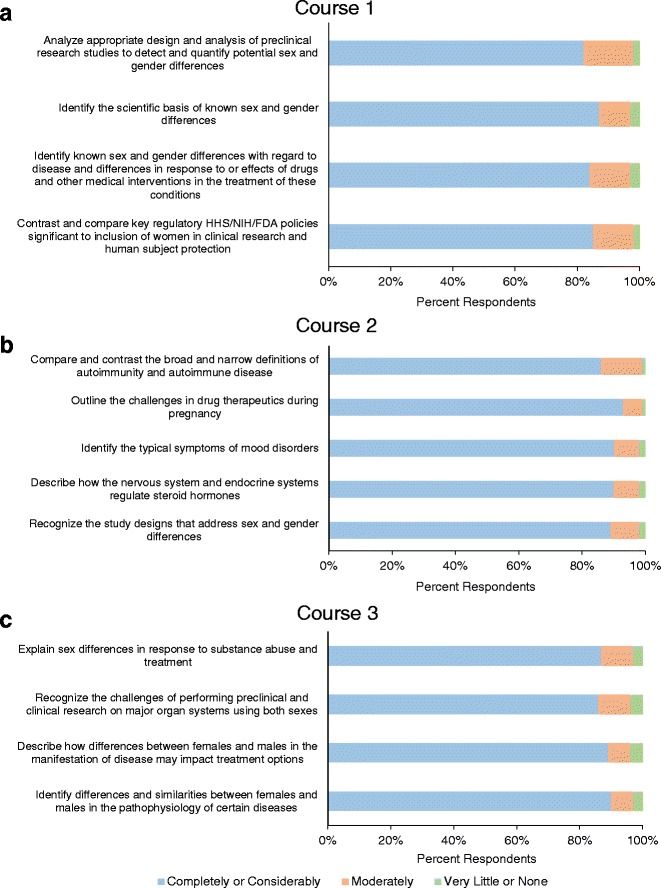
Percentage of user ratings of how well Course 1 (**a**), Course 2 (**b**), and Course 3 (**c**) met their objectives. Course completers indicated whether each course met its objectives, as indicated on the *y*-axis, considerably or completely (score of 4 or 5), moderately (score of 3), or very little or none (score of 1 or 2). The data indicate that the majority of the completers felt the courses met their objectives

The majority of the respondents also indicated that the courses considerably or completely met each of their objectives (Fig. 3). Respondents were asked to rate the extent to which a course met each of its objectives on a 5-point scale, with 5 being the highest score. For course 1, there was a range of 82 to 87 % of the respondents who indicated that the course considerably or completely (score of 4 or 5) met its objectives (Fig. 3a). Alternatively, only 2 to 3 % of the respondents indicated the course did not meet its objectives (score of 1 or 2). Similarly for course 2, 87 to 93 % of the participants responded with a score of 4 or 5, while 1 to 2 % of the participants responded with a score of 1 or 2 (Fig. 3b). Finally for course 3, 86 to 91 % of the respondents indicated that the course considerably or completely met its objectives, while 3 to 4 % said that it did not (Fig. 3c).

## Discussion

“The Science of Sex and Gender in Human Health” courses are unique in their advantages. The online nature allows users to complete the courses at their own pace, as time allows. Additionally, the courses are available at no cost to the user, allowing participants to obtain continuing education credits without a cost barrier. Finally, since they are not affiliated with a single academic institution, many renowned experts in understanding the science of sex and gender differences have provided content and lessons for the courses, ensuring the users are learning a broad-based perspective of state-of-the-science information. Since the launch of the courses, they have been widely viewed and completed by over 1000 users, suggesting that they have had an impact on the dissemination of information related to the influence of sex and gender in human health (Table 2). The positive, post-course surveys indicate overall user satisfaction with the courses (Fig. 3). Future courses should include a pre-test to facilitate evaluation of effectiveness and measure the attitudes of users in the post-course surveys. This additional information would be a valuable resource for improving the course content and design.

However, these findings do highlight considerable challenges. One observed trend is a decreasing number of registrants over time (Fig. 2). The decrease in number of registrants may be a result of interested users registering early and using the same registration information for subsequent courses, resulting in a lower number of new registrants. Another possibility is that the courses have not been adequately advertised. The NIH ORWH and the FDA OWH have done outreach, including social media, conferences, and other stakeholder engagement targeting a variety of health professionals including physicians, nurses, and pharmacists. This outreach targeted health professionals that may not specifically be involved in sex and gender research. Therefore, they may not want to take the courses for credit but, rather, to gain general knowledge about sex and gender issues. OWH and ORWH are currently broadening their approach for targeting individuals who may be interested in the courses.

There is also a significant gap between the number of registrants (4744, Fig. 2) and the number of course completers (1,223, Tables 1 and 2). There are several potential reasons for the low rate of completion. First, the courses can be time consuming, requiring approximately 6 h for completion. It is also possible that users are only interested in the content of a single course. Indeed, 2129 users, or 45 % of registrants, completed the first lesson of course 1, which is significantly higher than the overall completion rate (Tables 1 and 2). Completion credit was received only if all lessons in a course were completed. This may have actually discouraged, rather than encouraged, course completion because there was no achievement earned throughout the course. For this reason, it is important that future courses provide continuing education credit for each completed lesson, rather than the entire course. Finally, users who were not satisfied with the courses may have decided against completing them. Newly developed courses should attempt to close this gap.

Regardless of the reason for decreased registration or the low number of users completing courses, expertise should be garnered from education specialists and instruction designers to configure lesson content and structure for better learner engagement. For example, adult learners retain information best in short increments of time [16], which is in agreement with our findings that the average length of a visit to the website is just over 35 min. Therefore, future efforts to improve the courses will include decreasing the time needed to complete a lesson. Designing the platform to remember where a user left a lesson will also allow the user to complete lessons in segments, at their convenience. To increase learner interaction, questions can be added throughout the lesson rather than only at the end. Finally, developing a platform that can be viewed easily on mobile devices will increase the likelihood of lesson and course completion as users can more readily access the material.

Currently, the NIH ORWH and the FDA OWH are collaborating to develop course 4 in the series. This course will contain a mix of both basic and clinical science. In addition to the development of a new course, courses 1–3 will be updated to a new format that will also include a mobile-friendly design. The online nature of the courses provides an opportunity to readily update and improve the user experience.

The courses continue to be a valuable resource. They are comprehensive, offering a breadth and depth of subject matter and perspective. As a federally supported resource, they are free to the public. Furthermore, they are timely; in 2015, the NIH released a Guide Notice (NOT-OD-15-102) highlighting the expectation that sex as a biological variable will be factored into research designs, analyses, and reporting in vertebrate animal and human studies [17]. As the scientific community reaches for educational resources in sex- and gender-based research, “The Science of Sex and Gender in Human Health” online courses now stand to provide an even greater resource.

## Conclusions

In summary, we have provided an overview of the series of courses “The Science of Sex and Gender in Human Health” supported by the NIH Office of Research on Women’s Health and the FDA Office of Women’s Health. The courses are publicly available at no charge, and upon completion, users can receive continuing education credit.
